# Scaling up implementation of the E-MOTIVE bundle for early detection and management of postpartum haemorrhage emergencies: A scoping review

**DOI:** 10.4102/hsag.v31i0.3290

**Published:** 2026-09-30

**Authors:** Maurine R. Musie, Sibonginhlanhla P. Mdanda, Fhumulani M. Mulaudzi, Sinethemba Nyandeni, Adolphina M. Thema, Felicia O. Meno

**Affiliations:** 1 Department of Nursing Sciences, Faculty of Health Sciences, University of Pretoria, Pretoria, South Africa; 2 Ubuntu Community model in Nursing, Faculty of Health Sciences, University of Pretoria, Pretoria, South Africa

**Keywords:** E-MOTIVE-bundle, postpartum haemorrhage, obstetric emergencies and complications, maternal healthcare, EmONC, maternal mortality

## Abstract

**Background:**

Postpartum haemorrhage (PPH) remains a leading cause of maternal mortality globally. Effective management of PPH demands the implementation of E-MOTIVE bundle for early detection and management of PPH as recommended by the World Health Organization (WHO).

**Aim:**

The scoping review aimed to map evidence on the implementation of the E-MOTIVE bundle for early detection and management of PPH emergencies globally.

**Methods:**

The review adopted the Arksey and O’Malley methodological framework (2005). Six databases (CINAHL, MEDLINE, Scopus, PubMed, Web of Science and ProQuest) were searched for sources. The inclusion criteria consisted of full-text articles in English published between 2015 and 2025.

**Results:**

A total of 14 articles were selected. The review revealed: 1. Types of PPH bundles, 2. Implementation strategies of E-MOTIVE bundle. 3. Barriers to effective implementation of the E-MOTIVE bundle.

**Conclusion:**

A bundled care approach for the management of PPH following vaginal delivery has been shown to reduce adverse maternal outcomes because of PPH.

**Contribution:**

The review addresses a critical gap in the implementation and suggests implementation strategies of the E-MOTIVE bundle, including objective blood loss estimation, scalable training, adoption to national policies and teamwork among the obstetric team.

## Introduction

The World Health Organization (WHO 2012) initially released recommendations for the prevention and treatment of postpartum haemorrhage (PPH). Yet, more than a decade later, PPH continues to claim countless maternal lives, as many countries grapple with the persistent challenges to effectively implement strategies for early detection and management of PPH emergencies (Bohren et al. 2021). Postpartum haemorrhage is defined as a blood loss of ≥ 500 mLs in normal vaginal deliveries, and severe PPH is a blood loss of ≥ 1000 mLs after childbirth or caesarean section delivery in 24 h (Bohren et al. 2021, 2025; Forbes et al. 2023; Günaydın 2022). Postpartum haemorrhage remains one of the leading causes of preventable maternal mortality, contributing to approximately 27% of maternal deaths globally and between 30% and 50% of maternal deaths in sub-Saharan Africa supported by a Cochrane review conducted by Dwan et al. (2024), indicating that perinatal tears are a major contributor to PPH. In response to these persistent challenges, there is a growing body of literature that recognises the implementation of the WHO (2023b; 2025) strategies called E-MOTIVE bundle, designed to support the early detection and management of PPH following normal vaginal delivery (Forbes et al 2023; WHO 2023a). This bundle emphasises the use of a calibrated blood collection drape to enable timely detection, alongside evidence-based interventions delivered by skilled birth attendants within 15 min of identifying haemorrhage, to effectively control bleeding.

The causes of PPH include the 4 Ts uterine atony, birth canal trauma, retention of placental products and coagulation disorders (Petca et al. 2022; Yunas et al. 2025; Zenani et al. 2025). Bohren et al. (2025) highlight a particular concern: the most contributory factors to postpartum haemorrhage emergencies (PPH) are often preventable and avoidable causes. The initial factor includes late detection or missed identification of PPH post childbirth. The current status quo is that the obstetric team, comprising midwives and obstetricians, often uses ‘visual estimation’, observing visible pooling of blood under the woman, on the mattress and on soaked pads and linen as a method of estimating blood loss, which is often subjective, inaccurate and varies substantially between health providers (Bohren et al. 2021). The second factor is ineffective management of the PPH. The National Committee for Confidential Enquiries into Maternal Deaths (NCCEMD) in South Africa has emphasised that management failure of PPH includes late detection and substandard care ‘too little too late’ as the cause of mismanagement PPH (Bohren et al. 2025; Singata-Madliki et al. 2023). Bohren et al. (2025) further suggest that among the factors contributing to PPH, the WHO E-MOTIVE bundle, also known as Care Bundles for PPH in other contexts is poorly implemented, especially in low-income and middle-income countries (LMICs) because of limited knowledge and skills, workforce shortages, inadequate medication and supply stockouts.

To address the contributing factors, the WHO (2023) has recommended the E-MOTIVE bundle, which is a new ‘Care Bundles’ aimed at improving the detection and first-response management of PPH as suggested by the WHO technical consultation of PPH bundle development (Durocher & Ononge 2025). The E-MOTIVE bundle is set as a tool to be used during emergencies as the first-line response in maternity hospitals, including six evidence-based interventions (Bohren et al. 2025; Fawcus 2020). The interventions include (1) E- stands for early detection of PPH using a calibrated blood collection drape. (2) M- is for massaging of the uterus. (3) O- means oxytocic drugs (10 IU in 200 ml of normal saline over 5 min – 10 min followed by 20 IU in 1 L over 4 h – 8 h). (4) T- is for tranexamic acid (TXA) (1000 mg in 100 mL – 200 mL over 10 min) to be administered. (5) IVs are intravenous fluids to be given. (6) E- stands for the examination, i.e. looking for the source of bleeding and identifying it, and escalation refers to advanced care, if bleeding persists despite first response (Forbes et al. 2023). Care bundles have been associated with improved patient outcomes when adherence is high. The implementation of care bundle for PPH may include behaviours such as the widely used airway, breathing, circulation ‘ABCs,’ which were designed in emergency care to remind health professionals of the sequence of resuscitation (Althabe et al. 2020; Musie et al. 2025; Shikuku et al. 2022). The bundle approach has been proposed as a suitable solution to suboptimal adherence to PPH guidelines.

Despite the promising outcomes of the E-MOTIVE bundle, several gaps remain in the literature regarding its implementation. Previous studies reported on the need to change clinical behaviour for effective implementation of the bundle (Bohren et al. 2021; Forbes et al. 2023). Previous studies omitted to report on suggested strategies that may enhance the implementation of the E-MOTIVE bundles across different countries. Therefore, the review aims to map and synthesise evidence on the implementation of the E-MOTIVE bundle for early detection and management of PPH emergencies globally.

### Review question

The following is the review question: *What are the available implementation strategies of the E-MOTIVE bundle for early detection and management of postpartum haemorrhage (PPH) emergencies globally?*

## Methods

### Research design

This scoping review followed a five-step framework proposed by Arksey and O’Malley (2005) in Figure 1, and the reporting is done in accordance with the Preferred Reporting Items for Systematic Reviews and Meta-Analyses extension for scoping reviews (PRISMA-ScR) checklist.

**FIGURE 1:**
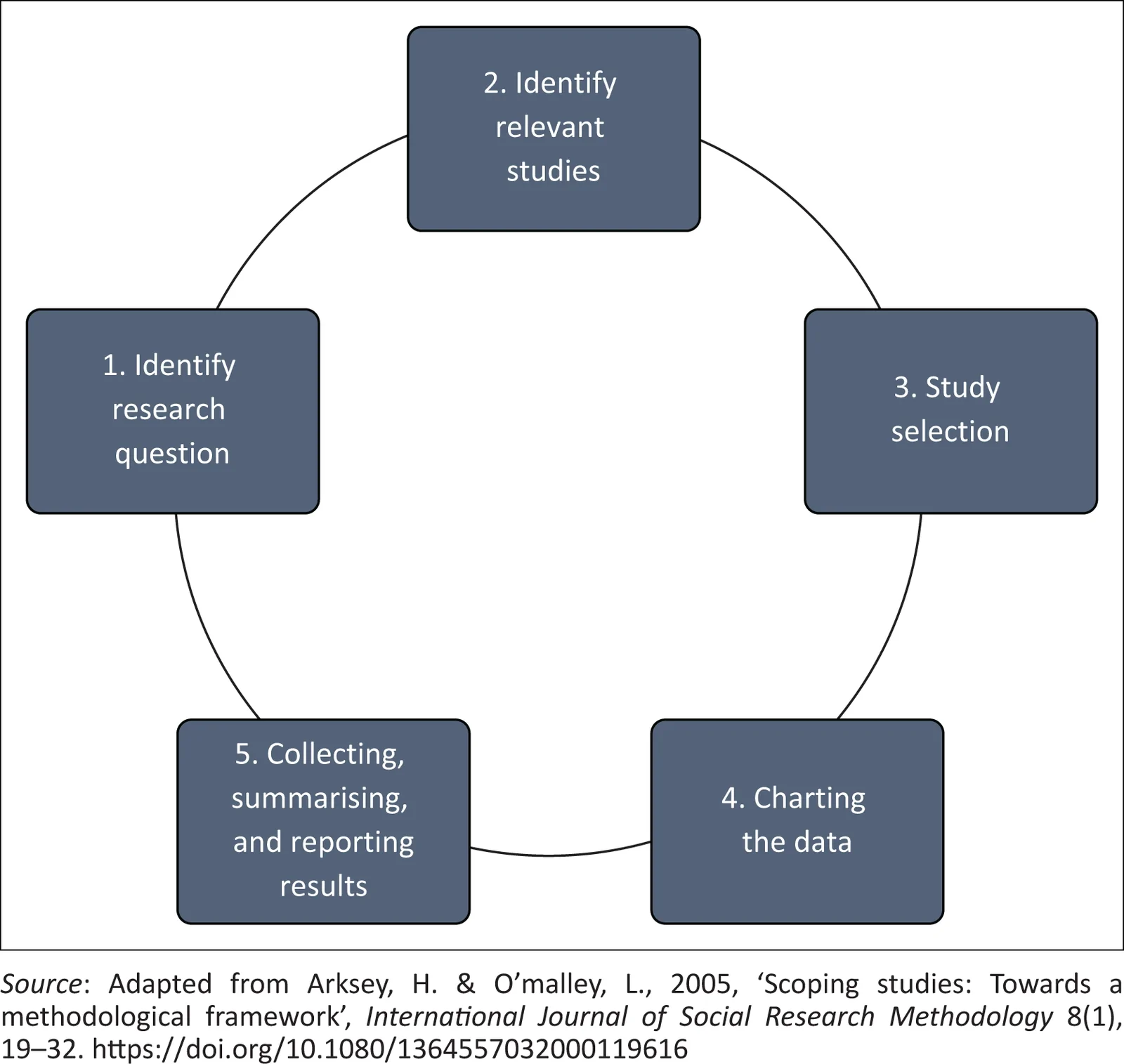
The five-stage framework for scoping reviews.

### Eligibility criteria

This scoping review collected and analysed peer-reviewed full-text empirical research articles written in English from 2015 to 2025 to ensure the latest articles pertaining to the implementation of the E-MOTIVE bundle globally are included. The scoping review followed the Population, concept and context (PCC) framework (Peters et al. 2021); inclusion and exclusion criteria were formatted (Table 1).

**TABLE 1:** Inclusion and exclusion criteria based on population, concept and context.

Terms	Inclusion criteria	Exclusion criteria
Population	Obstetric team comprising midwives, nurses, obstetricians and gynaecologists responsible for implementing the E-MOTIVE bundle	Health professionals not included in obstetric team
Concept	E-MOTIVE bundle or care bundle or PPH bundle or bundle strategy	Any other studies not referring to postpartum haemorrhage
Context	Globally Studies from 2015 to 2025	-

PPH, postpartum haemorrhage.

### Search strategy, selection of sources and data extraction

The search strategy was developed in consultation with the ‘Information specialist redacted to maintain the integrity of the review process ‘Information specialist and the input of the research team. The authors searched for the relevant studies on the following six databases: CINAHL, MEDLINE, Scopus, PubMed, Web of Science and ProQuest, to retrieve the most relevant data. Descriptions such as title, abstract and full-text screening of the relevant and suitable studies on the topic of interest were used to conduct the search strategy. The following medical subject heading (MeSH) terms were used: e.g. (‘E-MOTIVE Bundle’ OR ‘EMOTIVE Bundle’ OR ‘bundle strategy’ OR ‘Care bundle’) And (‘Obstetricians’ OR ‘Midwives’ OR ‘Maternity’ OR ‘Obstetric’ OR ‘on-gyn’ OR ‘Gynaecologist’ OR ‘Midwife’) AND (‘Detection’ OR ‘Management’) AND (‘Postpartum haemorrhage’ OR ‘Haemorrhage’ OR ‘haemorrhage’ OR ‘Immediate Postpartum’ OR ‘Delayed postpartum’).

Following the literature search, all the identified studies were imported to the systematic review management system, namely Covidence (2023), which guided the screening process, title and abstract screening, review of full-text and data extraction. The eligibility for inclusion of articles was assessed independently by two reviewers (first author and second author). When disagreement or conflicts arise with the included or excluded articles, the two reviewers met and reached consensus for inclusion, and it was checked by the third author. The search results and decisions at each stage of extraction are summarised by the PRISMA ScR flowchart to illustrate the search process (refer Figure 2). Using a data extraction tool in the software, which was later extracted to an Excel sheet. The data were extracted by a researcher (refer Table 2). No formal quality assessment of the articles was conducted, as scoping reviews typically do not require an evaluation of methodological quality.

**FIGURE 2:**
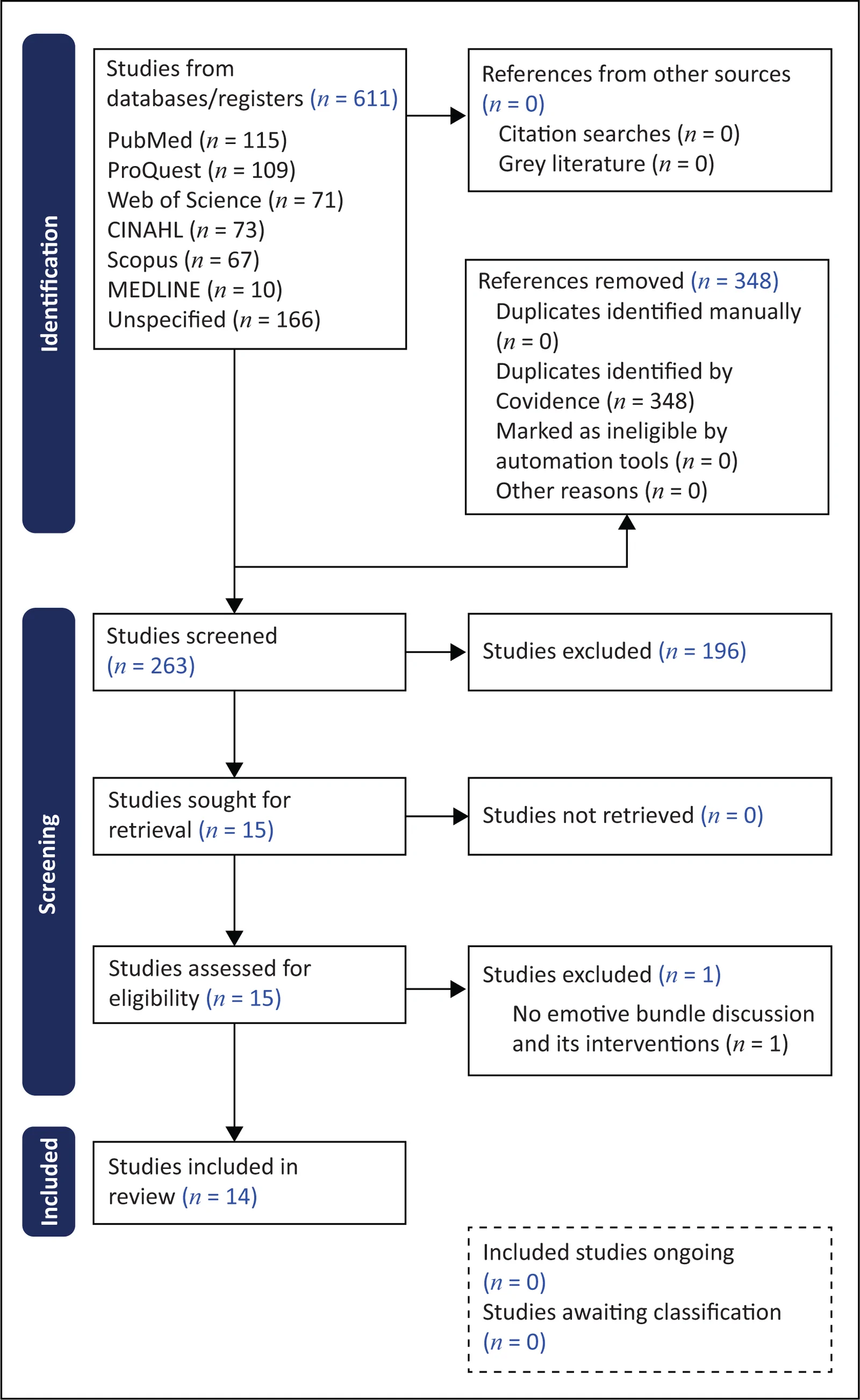
Preferred Reporting Items for Systematic Reviews and Meta-Analyses extension for scoping reviews flowchart illustrating the search process.

**TABLE 2:** Characteristics of the included studies.

Author and Year	Title	Objectives	Population (P) and Context (C)	Research design	Results	Implementation strategy*
1. Bohren et al. (2025)	Early detection and a treatment bundle strategy for postpartum haemorrhage: a mixed-methods process evaluation	Assessing the implementation of the E-MOTIVE intervention in Kenya, Nigeria, South Africa and Tanzania	P = Midwives, nurses, doctors, pregnant women. C = Kenya, Nigeria, South Africa and Tanzania	Mixed method design (MMD) using cluster-randomised trial	After the implementation of the E-MOTIVE bundle for 7 months, the study reported that *n* = 295 women experienced postpartum haemorrhage (PPH). In the hospital, the health professionals used the calibrated drapes for blood collection (100%).	The primary implementation outcomes include fidelity, adoption, adaptation, acceptability, feasibility and contamination to the calibrated drape, E-MOTIVE bundle and implementation strategies. *Implementation strategy = E-MOTIVE bundle
2. Forbes et al. (2023)	Factors influencing postpartum haemorrhage detection and management and the implementation of a new postpartum haemorrhage care bundle EMOTIVE in Kenya, Nigeria and South Africa	Explore influences on current PPH detection and management and on the future implementation of a new PPH bundle (E-MOTIVE) in low-resource, high-burden settings	P- *n* = 45 Healthcare providers across nine hospitals. C-Nigeria, Kenya and South Africa.	Qualitative research design	The results highlight that PPH detection and management were influenced by 12 domains of the theoretical domains framework (TDF)	The study proposed five implementation interventions: 1. The use of Calibrated Drape 2. E-MOTIVE training increasing competencies on PPH management, 3. E-MOTIVE champions, 4. PPH emergency trolley 5. Audit and feedback. *Implementation strategy = E-MOTIVE bundle
3. Williams et al. (2024)	A cost-effectiveness analysis of early detection and bundled treatment of postpartum haemorrhage alongside the E-MOTIVE trial	To assess the cost-effectiveness of the E-MOTIVE intervention, which included a calibrated blood collection drape for early detection of PPH and a bundle of first-response treatments	P- *n* = 210, 132 women undergoing vaginal delivery across 78 secondary-level hospitals. C = Kenya (14) Nigeria (38) South Africa (14) Tanzania (12)	Parallel cluster-randomized trial (RCT)	There was improved detection of PPH facilitated by the use of a calibrated blood collection drape and consequent triggering of the bundle.	Four strategies can be used for implementation of the E-MOTIVE bundle in the hospital. 1. Audit newsletters 2. Champions 3. Trolley or carry case 4. Training: on-site, simulation-based *Implementation strategy = E-MOTIVE bundle
4. De Tina et al. (2019)	Identifying Barriers to Implementation of the National Partnership for Maternal Safety Obstetric Haemorrhage Bundle at a Tertiary Center: Utilization of the Delphi Method	To identify practice deficiencies and perceived barriers to bundle implementation among multidisciplinary providers	p= population A total of 38 experts completed the study (11 anaesthesiologists, 11 obstetricians, 10 nurses, and 6 surgical technicians). C = United States	Prospective, cross-sectional, consensus-building study based on the Delphi method.	The results revealed four barriers to implementation were identified: (1) medications not being stored in the operating room; (2) the electronic medical record limiting resource readiness (3) staffing, patient care and shift limitations preventing consistent huddles and debriefs and (4) surgical sponges or laparotomy pads not being routinely weighed or cumulatively recorded.	Six Strategies recommended for implementation feasibility. 1. Immediate access to haemorrhage medications 2. Unit education on protocols, unit-based simulation drills with post-drill debriefs 3. Assessment of haemorrhage risk- blood loss quantification 4. Unit-standard, stage-based obstetric haemorrhage emergency plan with checklists 5. Support programme for patients 6. Establish a culture of huddles for high-risk patients *Implementation strategy = National Partnership for Maternal Safety (NPMS) developed an obstetric haemorrhage consensus bundle.
5. Joseph et al. (2020)	Implementation of a Postpartum Haemorrhage Safety Bundle at an Urban Safety-Net Hospital	The primary objective is to describe the methodology for programme implementation of the Alliance for Innovation on Maternal Health Safety Bundle on PPH at an urban safety-net hospital.	P, C = United States	Case report- *Plan-Do-Study-Act* (PDSA) cycles	Haemorrhage risk assessment stratification rates improved to > 90% during bundle implementation.	1. Standardise a haemorrhage protocol 2. Define a haemorrhage response team and ensure ongoing education on haemorrhage management for all providers on the unit, 3. Routinise use of the Postpartum Haemorrhage Risk Assessment Tool, 4. Employ quantitative blood loss (QBL) for all deliveries, 5. Develop and utilise haemorrhage carts, 6. Streamline protocol adherence with an obstetric narrator *Implementation strategy = Postpartum Haemorrhage Risk Assessment Tool (PHRAT)
6. Akter et al. (2023)	Perceptions and experiences of the prevention, detection and management of postpartum haemorrhage: a qualitative evidence synthesis (Review)	To explore the perceptions and experiences of women, community members, lay health workers, and skilled healthcare providers who have experience with PPH or with preventing, detecting, and managing PPH, in community or health facility settings.	P, C = Studies from low- or middle-income countries (33 studies), and most included the perspectives of women and health workers.	Most were from low- or middle-income countries (33 studies), and most included the perspectives of women and health workers.	The first responders to PPH are not always skilled healthcare providers. Inconsistent availability of resources (drugs, medical supplies, blood) causes delays in the timely management of PPH (high confidence).	Overall, factors associated with successful implementation (‘making a difference’) included 1. existing workplace cultures of safety and reflective learning, local PPH champions and positive attitudes. *Implementation strategy = E-MOTIVE bundle
7. Akter et al. (2022)	Detection and management of postpartum haemorrhage: Qualitative evidence on healthcare providers’ knowledge and practices in Kenya, Nigeria and South Africa.	To explore healthcare providers’ knowledge and practices of PPH detection and management after vaginal birth to inform design and implementation of E-MOTIVE.	P-Maternity healthcare providers (midwives, nurses, doctors, managers). C- Kenya, Nigeria and South Africa. Nine hospitals in Kenya, Nigeria and South Africa.	Qualitative research design	Four themes emerged. In-service training on emergency obstetric care; limited knowledge about PPH; current approaches to PPH detection and current PPH management and associated challenges.	The following strategies were used for implementation: Capability, Opportunity, Motivation and Behaviour (COM-B) and theoretical domains framework (TDF). 1. Improve in-service training 2. Objective measurement of blood loss 3. Leadership, teamwork, feedback and communication, Implementation strategy = E-MOTIVE bundle
8. Bingham, Scheich and Bateman (2018)	Structure, Process and Outcome Data of AWHONN’ Postpartum Haemorrhage Quality Improvement Project	To describe the structures and processes implemented during the Association of Women’s Health, Obstetric, and Neonatal Nurses Postpartum Haemorrhage (AWHONN PPH) Project	P-Volunteer registered nurse hospital leaders C-58 hospitals located in Washington, DC; Georgia and New Jersey. United States	An 18-month, multiregional, multihospital quality improvement project.	All structures and processes showed improvement but were not fully implemented at all sites. Registered nurse participation in drills increased from 0% to 92%, quantification of blood loss increased from 5% to 45%, haemorrhage risk assessment increased from 10% to 70%.	AWHONN PPH bundle
9. Main et al. (2015)	National Partnership for Maternal Safety Consensus Bundle on Obstetric Haemorrhage.	To pilot the development of a Consensus Bundle on Obstetric Haemorrhage	United States	Case report	The four domains of the safety bundle are readiness, recognition and prevention, response and reporting and system learning.	The safety bundle is organised into 13 domains Consensus PPH bundle. *Implementation strategy = National Partnership for Maternal Safety (NPMS) developed an obstetric haemorrhage consensus bundle.
10. Pingray et al. (2024)	Strategies for optimising early detection and obstetric first response management of postpartum haemorrhage at caesarean birth: a modified Delphi-based international expert consensus	The study aimed to develop an international expert consensus on evidence-based approaches for early detection and obstetric first-response management of PPH intraoperatively postoperatively in caesarean.	P-Panel of 22 global experts in PPH with diverse backgrounds and gender, professional and geographic balance. C-Global	Systematic review and three-stage modified Delphi expert consensus.	The experts reached consensus that a standardised definition of postpartum haemorrhage (PPH) should be applied to both vaginal and caesarean births. Furthermore, the experts agreed that the initial response to suspected PPH should include prompt identification of the underlying cause, immediate administration of uterotonics and tranexamic acid, and rapid initiation of cause-specific management.	The bundle approach for caesarean section includes 1. Definition of PPH for c-section is the same as for vaginal delivery. 2. The intraoperative early detection of PPH includes the quantitative measurement and ongoing assessment of woman’s haemodynamic status. 3. First response to PPH includes infusion of oxytocin. Tranexamic acid (TXA) within 30 min.
11. Tancred et al. (2025)	Assessment of Structural and Process Readiness for Postpartum Haemorrhage Care in Uganda and Ghana: A Mixed Methods Study	To determine structural and process readiness for postpartum haemorrhage (PPH) care at referral-level facilities in Ghana and Uganda to identify opportunities for strengthening	P, C-Nine hospitals in Ghana and seven in Uganda.	Mixed-methods situational analysis	In both countries, there was generally good structural readiness for PPH care. However, key common gaps included inadequate staffing (especially specialist physicians), and unavailability of blood for transfusion.	WHO (2017) Guidelines for PPH care 1. Availability of Standards and Protocols on site 2. Staffing and Team working 3. Availability of resources such as blood transfusions
12. Reale, Leffert and Farber (2019)	Update on Obstetric Haemorrhage	To provide the most recent epidemiologic data on postpartum haemorrhage in the United States	P, C = United States	Review	The use of a management protocol for PPH is a key recommendation of the NPMS haemorrhage bundle. Delayed recognition of PPH and ‘too little done too late’ continues to be an area of focus to reduce preventable morbidity.	*National Partnership for Maternal Safety (NPMS) consensus bundle on obstetric haemorrhage Areas of focus include standardising postpartum haemorrhage management with protocol use, massive transfusion protocols
13. Althabe et al. (2020)	Postpartum haemorrhage care bundles to improve adherence to guidelines: A WHO technical consultation	To systematically develop evidence-based bundles of care of postpartum haemorrhage (PPH)	P, C = Thirty-three global maternal-health experts	Clinical study	The consultation led to the definition of two care bundles for facility implementation. The ‘First response to PPH bundle’. The ‘Response to refractory PPH bundle’ comprises compressive measures	*WHO’s (2012 & 2017) PPH recommendations – First response to PPH bundle and response to refractory PPH bundle
14. Singata-Madliki et al. (2023)	Implementing E-MOTIVE for detection and treatment of postpartum haemorrhage in South Africa	Exploring the implementation of the E-MOTIVE for detection and treatment of postpartum haemorrhage in South Africa	P, C = Review 78 hospitals in Nigeria, Kenya, Tanzania and SA (14 hospitals in Western Cape, KwaZulu-Natal and Eastern Cape provinces)	Parallel cluster randomised trial (RCT)	The E-MOTIVE approach comprises objective blood loss measurement monitored every 15 min during the first hour after delivery to detect PPH early and trigger a bundle of first-line treatments.	Implementation of the E-MOTIVE intervention was facilitated by: 1. Provision of a ‘PPH box’ (except oxytocin) 2. Wall posters demonstrating the E-MOTIVE intervention 3. ‘Essential Steps in Managing Obstetric Emergencies’ (ESMOE) 4. Accurate determination of blood loss 5. Hospital E-MOTIVE signed protocols by senior doctors. 6. E-MOTIVE champions *Implementation strategy = E-MOTIVE bundle

Note: Please see full reference list of this article: Musie, M.R., Mdanda, S.P., Mulaudzi, F.M., Nyandeni, S., Thema, A.M. & Meno, F.O., 2026, ‘Scaling up implementation of the E-MOTIVE bundle for early detection and management of postpartum haemorrhage emergencies: A scoping review’, *Health SA Gesondheid* 31(0), a3290. https://doi.org/10.4102/hsag.v31i0.3290 for more information.

* , Refers to the type of implementation strategy used in the different studies of PPH bundle.

### Collating, summarising and reporting of results

In this step, the authors collated and summarised the results and drew conclusions from the review. Thematic analysis was performed by the first author according to Braun & Clarke (2006). The themes and subthemes on the implementation strategies of the E-MOTIVE bundle were identified.

### Ethical considerations

Ethical clearance to conduct this study was obtained from the University of Pretoria Faculty of Health Sciences Research Ethics Committee on 12 June 2025. The ethical approval number is 65/2025.

## Review findings

### Selection of sources

A total of 611 records were retrieved from 2015 to 2025 (Figure 2). Following the removal of 348 duplicates, 263 studies remained for screening. The following 196 articles were excluded for not meeting the eligibility criteria based on abstract and title screening. Only *n* = 15 articles were eligible for full-text review, and one article was excluded as no E-MOTIVE bundle implementation was mentioned. After the full-text screening, only *n* = 14 studies were included for review.

#### Theme 1: Classification of postpartum haemorrhage care bundles

**E-MOTIVE bundle:** The studies included report on a variety of PPH care bundles implemented globally for the early detection and management of PPH. According to Atlhabe et al. (2020) and Tancred et al. (2023), the ‘care bundles’ were developed from the (WHO 2012, 2017) recommendation for PPH focusing the first response to PPH bundle (comprising uteronics, isotonic crystalloids, tranexamic acid and uterine massage for immediate and basic interventions for PPH) and the response to refractory PPH bundle (management when PPH does not respond to first-line measures including aortic or bimanual uterine compression), the non-pneumatic anti-shock garment (NASG) and intrauterine balloon tamponade (IBT) measures. The first WHO Recommendations and guidelines on the prevention and management of postpartum haemorrhage (PPH) were published in (WHO, 2012), followed by updated recommendations in 2017. The current approach builds on these recommendations, with the 2023 WHO E-MOTIVE bundle providing an updated, evidence-based approach for the early detection and timely management of postpartum haemorrhage. The review highlights six studies (*n* = 6, 43%), which report on the implementation of the new released care bundle by the WHO (2023) called the E-MOTIVE bundle, which is evidence-based approach for early detection and treatment of PPH including the following sub-Saharan Africa countries: Kenya, Nigeria, South Africa and Tanzania and globally (Akter et al. 2022, 2023; Bohren et al. 2025; Forbes et al. 2023; Singata-Madliki et al. 2023; Williams et al. 2024), including massaging the uterus, oxytocin infusion, tranexamic acid infusion, intravenous crystalloid fluids, examination for the cause, emptying the bladder and, if necessary, escalation of care. In South Africa, the E-MOTIVE was integrated into the existing Essential Steps in Managing Obstetric Emergencies (ESMOE) algorithm.

**National Partnership for Maternal Safety consensus bundle on obstetric haemorrhage:** The review highlights three different types of care bundles of PPH utilised in the United States and other countries internationally. The first type of bundle is called the National Partnership for Maternal Safety (NPMS) consensus bundle on obstetric haemorrhage, implemented and reported in (*n* = 3, 21%) studies in countries such as the United States (De Tina et al. 2019; Main et al. 2015; Reale et al. 2019). The bundle was developed with the aim of reducing PPH mortality and morbidity. The NPMS is embedded in the following four domains: (i) readiness, (ii) recognition and prevention, (iii) response (iv) reporting and system learning.

**Association of Women’s Health, Obstetric and Neonatal Nurses postpartum haemorrhage bundle:** One (*n* = 1, 7%) study reported on the AWHONN PPH bundle, which refers to the set of evidence-based practices developed by the Association of Women’s Health, Obstetric and Neonatal Nurses (AWHONN) for the prevention and management of PPH (Bingham et al. 2018). This bundle is developed based on similar four domains as the NPMS bundle.

**Postpartum Haemorrhage Risk Assessment Tool:** In the United States, one (*n* = 1, 7%) study reported on the PHRAT, which is utilised to identify women at risk of PPH and prompt immediate interventions for management. The tool also includes standardisation of protocols for PPH and employs quantitative blood loss (QBL) for all deliveries (Joseph et al. 2020).

**Bundle of postpartum haemorrhage care for caesarean deliveries:** One study (*n* = 1, 7%) conducted globally revealed a bundle approach for the management of PPH for caesarean deliveries (Pingray et al. 2024).

#### Theme 2: Implementation strategy of the E-MOTIVE bundle

Most of the studies propose several implementation strategies of the E-MOTIVE bundle to address the detection and management of PPH emergencies. These strategies will be discussed under the four R- Readiness, Recognition and Prevention, Response and Reporting and Systems Learning.

**Recognition and prevention: Use of calibrated drapes:** Most of the studies report the use of the calibrated drape as the first tool according to the E- for early identification of PPH within the first 15 min of recognising haemorrhage to initiate rapid response interventions. The drape includes measurements that allows for QBL objective assessment of blood loss after every vaginal birth (Akter et al. 2022; Joseph et al. 2020; Reale et al. 2019; Tancred et al. 2025) as described according to the National Partnership for Maternal Safety (NPMS) consensus bundle.The health provider uses a plastic calibrated blood collection drape placed under the woman’s buttocks to measure the amount of blood loss, addressing the delays in PPH detection and inaccurate measurement of blood loss (Akter et al. 2022, 2023; Bohren et al. 2025; Forbes et al. 2023; Singata-Madliki et al. 2023; Williams et al. 2024).

**Readiness: Postpartum haemorrhage emergency trolley and/or kit to promote appropriate postpartum haemorrhage care provision:** The readiness to manage the PPH emergency can be ensured through implementation strategies consisting of PPH trolleys or carry cases with necessary medications and supplies (except for oxytocin, which requires refrigeration). Furthermore, Tancred et al. (2025) further add that the trolleys should include medications as stipulated on the national standard protocols. A study done in South Africa adds that these carry cases is called the PPH box, which consist of all necessary equipment for the emergency and should be implemented within 15 min of diagnosing PPH (Singata-Madliki et al. 2023).

**E-MOTIVE champions:** Another strategy that has been suggested is the introduction of E-MOTIVE champions in every maternity units, which may assist to encourage the health professionals who may be resistantto initiate the E-MOTIVE bundle (Forbes et al. 2023). These champions will act as a response team as day-to-day leaders, ensuring the implementation of evidence-based interventions for management of PPH.

**Response: E-MOTIVE training increasing competencies on postpartum haemorrhage management:** The third identified implementation strategy includes training the obstetric team on the new E-MOTIVE bundle for management of PPH. Most of the studies (*n* = 8) suggested the training to be done on how to use the calibrated drape for blood collection (Akter et al. 2022, 2023; Bohren et al. 2025; Forbes et al. 2023; Singata-Madliki et al. 2023; Williams et al. 2024). The health workers working at the maternity units may undergo continuous professional development and attend onsite workshops on simulation-based such as drills on PPH, include flip-charts of the E-MOTIVE displayed in the delivery rooms (Williams et al. 2024). A study conducted in South Africa also suggests the inclusion of the E-MOTIVE bundle into the national policies for different countries (Singata-Madliki et al. 2023).

**Reporting and systems learning: Audit and feedback:** The audit and feedback strategy is identified under the Capability, Opportunity, Motivation and Behaviour (COM-B) and theoretical domains framework (TDF) (Akter et al. 2022). As supported by Main et al. (2015) which indicates the 13 domains for effective implementation of the PPH bundles. Under the last domain entitled Reporting & Systems Learning (Every unit), it is expected that each maternity unit conducts monthly departmental meetings where cases such as PPH are presented and critically discussed.

#### Theme 3: Barriers to implementation of the E-MOTIVE bundle

**“Too little too late” (TLTL) interventions:** The overarching barrier that was identified in the reviews is that the Too Little too late’ interventions is the barrier to effective implementation of the E-MOTIVE bundle. This refers to delayed, inadequate or incomplete responses of PPH that fail to prevent progression to severe morbidity or maternal death. The first one most cases the health providers delayed recognition of PPH by failing to quantify blood loss (Reale et al. 2019). The second one is failure to administer the first-line treatment of the E-MOTIVE bundle, which includes massaging the woman’s uterus and uterotonics not administered promptly or given in insufficient doses. The last delay is poor Referral and Escalation Systems, as seen in a study indicating clear gaps in the utilisation of tranexamic acid and uterine balloon tamponade not in line with the WHO guidelines for PPH management (Tancred et al. 2025).

## Discussion

This review shows that the implementation of the WHO (2023b) E-MOTIVE bundle is associated with lower rates of adverse outcomes compared to usual care for PPH management. It is also associated with improved detection of PPH and a high rate of implementation of evidence-based interventions. The E-MOTIVE bundle consisted of a calibrated blood collection drape for early detection of PPH (E) and a bundle of first-response treatments (MOTIVE) comprising uterine massage, oxytocic drugs, tranexamic acid, intravenous fluids, examination and escalation (Bohren et al. 2025; Devall et al. 2025; Williams et al. 2024).

The review revealed the implementation in four African countries, an important consideration for future scale-up of the E-MOTIVE bundle is the quantity and quality is the need for adoption and implementation of the bundle in all countries globally (Durocher et al. 2025). On a global scale, maternal morbidity and mortality are predominantly associated with obstetric emergencies, including ectopic pregnancy, placental abruption, placenta previa, preeclampsia or pregnancy-induced hypertension, eclampsia, premature rupture of membranes (PROM), amniotic fluid embolism (AFE), uterine inversion or rupture, placenta accreta, umbilical cord prolapse and shoulder dystocia. Within this spectrum, PPH persists as the second leading cause of maternal mortality worldwide (WHO 2022; Zenani et al. 2025). These obstetric emergencies hinder progress towards the achievement of Sustainable Development Goal (SDG) 3.1, which aims to reduce the global maternal mortality ratio (MMR) to fewer than 70 deaths per 100 000 live births by 2030 (United Nations Assembly 2015).

The review calls for scaling up the implementation of the E-MOTIVE bundle for early detection and management of PPH. For effective implementation, interventions need to be adapted and scaled up using the Capability, Opportunity, Motivation, and Behaviour (COM-B) model in conjunction with the TDF (Akter et al. 2023).

Barriers to effective implementation have also been identified in countries such as Nigeria, Kenya and South Africa, noting several factors that may influence the implementation of the E-MOTIVE bundle, such as that the bundle favours a supine flat position for the drape to collect the blood loss (Forbes et al. 2023). A study reported nine cases of maternal near-miss because of PPH, corresponding to a maternal near-miss ratio (MNMR) of 8.7 per 1000 live births, with severe PPH contributing to 35% of maternal deaths (Mulongo, Kaura & Mash 2024). There is little belief that tranexamic acid can be used in the management of PPH as it was not used before. Therefore, midwives have no confidence in its application. Information on the use of the bundle is poor to the obstetric team; more training is expected so that the team can be oriented and skilled. This prevents the proper implementation of the bundle. The bundle comprises the various principles of PPH management that were used individually; some midwives are still dependent on the previous practice instead of following the whole bundle. Therefore, the statistics for PPH are still high because of barring factors to this E-MOTIVE bundle (Forbes et al. 2023). These comparisons emphasise the urgent need for standardised, evidence-based protocols such as E-MOTIVE to bridge critical gaps in implementation of the E-MOTIVE bundle for PPH management globally.

### Strengths and limitations

A key strength of this scoping review is that it is the first to specifically examine implementation strategies for the E-MOTIVE bundle at a global level. However, a limitation is that the review was restricted to published literature, which may have excluded relevant grey literature and unpublished implementation experiences, potentially introducing bias.

## Conclusion

This review mapped the existing evidence on the implementation of the E-MOTIVE bundle for the early detection and management of PPH emergencies globally. Countries may draw lessons from South Africa, where the E-MOTIVE bundle has been integrated into the ESMOE (Essential Steps in Managing Obstetric Emergencies) training for healthcare professionals and incorporated into national guidelines. For effective scale-up, this review recommends that obstetric teams undergo capacity building to enhance healthcare providers’ knowledge, skills and confidence through targeted training, simulation drills and continuous professional development programmes for postpartum management.
